# The definitions, assessment, and dimensions of cancer-related fatigue: A scoping review

**DOI:** 10.1007/s00520-024-08615-y

**Published:** 2024-06-25

**Authors:** Kayla F. Keane, Jordan Wickstrom, Alicia A. Livinski, Catherine Blumhorst, Tzu-fang Wang, Leorey N. Saligan

**Affiliations:** 1 National Institute of Nursing Research, National Institutes of Health, Bethesda, MD USA; 2 Sinai Rehabilitation Center, Sinai Hospital of Baltimore, Baltimore, MD USA; 3 Rehabilitation Medicine Department, Clinical Center, National Institutes of Health, Bethesda, MD USA; 4 Office of Research Services, Office of the Director, National Institutes of Health Library, National Institutes of Health, Bethesda, MD USA

**Keywords:** Cancer-related fatigue, Fatigue definition, Fatigue dimension, Fatigue assessment, Scoping review

## Abstract

**Purpose:**

Cancer-related fatigue (CRF) is challenging to diagnose and manage due to a lack of consensus on its definition and assessment. The objective of this scoping review is to summarize how CRF has been defined and assessed in adult patients with cancer worldwide.

**Methods:**

Four databases (PubMed, Embase, CINAHL Plus, PsycNet) were searched to identify eligible original research articles published in English over a 10-year span (2010–2020); CRF was required to be a primary outcome and described as a dimensional construct. Each review phase was piloted: title and abstract screening, full-text screening, and data extraction. Then, two independent reviewers participated in each review phase, and discrepancies were resolved by a third party.

**Results:**

2923 articles were screened, and 150 were included. Only 68% of articles provided a definition for CRF, of which 90% described CRF as a multidimensional construct, and 41% were identical to the National Comprehensive Cancer Network definition. Studies were primarily conducted in the United States (19%) and the majority employed longitudinal (67%), quantitative (93%), and observational (57%) study designs with sample sizes ≥ 100 people (57%). Participant age and race were often not reported (31% and 82%, respectively). The most common cancer diagnosis and treatment were breast cancer (79%) and chemotherapy (80%; *n* = 86), respectively. CRF measures were predominantly multidimensional (97%, *n* = 139), with the Multidimensional Fatigue Inventory (MFI-20) (26%) as the most common CRF measure and “Physical” (76%) as the most common CRF dimension.

**Conclusion:**

This review confirms the need for a universally agreed-upon definition and standardized assessment battery for CRF.

**Supplementary Information:**

The online version contains supplementary material available at 10.1007/s00520-024-08615-y.

## Introduction

More than 1.9 million Americans are diagnosed with cancer each year [1], with 80–100% experiencing cancer-related fatigue (CRF) as a result of the disease and/or treatment [2–4]. Fatigue experienced by those with cancer (i.e., CRF) differs from fatigue experienced by healthy individuals by its severity, chronic impact on quality of life, persistence (often lasting more than six months), and inability to be resolved by sleep or rest [2, 4, 5]. In addition, CRF also imposes a significant burden on employment and finances, not only for people with a cancer diagnosis but also for their families [6, 7].

Currently, there is no gold standard for how to define CRF [8], but several organizations have proposed definitions, including the National Comprehensive Cancer Network (NCCN) [9], the European Society for Medical Oncology [10], American Society of Clinical Oncology [11], and the Pan Canadian Practice Guidelines [12]. These definitions share common elements in that they describe CRF as a subjective exhaustion brought on by cancer or its treatment that interferes with daily activities, disproportionate to the level of exertion, and is not relieved by rest. Despite the availability of multiple definitions, the NCCN definition is most often used [8], defining CRF as “a distressing, persistent, subjective sense of physical, emotional, and/or cognitive tiredness or exhaustion related to cancer or cancer treatment that is not proportional to recent activity and interferes with usual functioning” [9]. A previous review found that some articles reference diagnostic criteria, such as criteria from the National Cancer Institute Common Toxicity or the International Statistical Classification of Diseases and Related Health Problems (ICD-10), to define CRF [13] and others fail to report a definition altogether. The absence of a single agreed-upon definition contributes to the challenge of assessing (let alone treating) CRF, but working toward a standard definition should be a primary focus of the field.

Since CRF is a subjective, multidimensional construct, numerous questionnaires have been used to assess CRF, with no consensus on a battery of assessments to characterize its dimensions. In support of this notion, a recent scoping review of intervention studies revealed the use of 58 unique CRF measures [14], confirming the significant challenge to determine which assessments are most appropriate. Efforts to develop clinical practice guidelines recommending screening and assessment methods for CRF are ongoing. One such effort is the clinical practice guideline from the American Physical Therapy Association which recommended Grade A (high level of certainty) screening and assessment tools for CRF [15]. Guidelines such as these are needed to help standardize our approach to screening and assessing CRF, inching us closer to understanding the etiology of CRF and identifying effective management solutions. Therefore, this scoping review contributes to these efforts by exploring and elucidating the complex, multidimensional nature of CRF through the following objective and specific aims.

- Objective:

- To determine how CRF has been defined and assessed across studies in adults with cancer worldwide.

- Specific Aims:
- Aim 1 – Describe how CRF has been defined, including

- Proportion of articles that define CRF, and of these articles;
- Proportion that use a multidimensional definition of CRF;
- Proportion that use the NCCN definition.

- Aim 2 – Determine how CRF has been assessed, including:

o Proportion of articles that use multidimensional clinical measures;
o Most common CRF measures used;
o Most common CRF dimensions assessed.

- Aim 3 – Characterize the articles reviewed, including:

o Where (countries) the studies were conducted;
o Demographics of the samples enrolled in the studies;
o Cancer diagnoses, treatments, and interventions that were included;
o Study designs used.

## Methods

We followed the scoping review methods from the Joanna Briggs Institute [16] and used the Preferred Reporting Items for Systematic Reviews and Meta-Analyses Extension for Scoping Reviews (PRISMA-ScR) for the reporting of this review [17].

### Protocol

We used the PRISMA-ScR as an outline for writing an a priori protocol.

### Eligibility criteria

Full-length original research articles published in English between January 2010 and December 2020 that contained human adult participants (18 years and older) with a cancer diagnosis (as defined by the National Cancer Institute) [18] were included in this review. CRF was required to be a primary outcome of interest and be described in terms of how it was assessed. Detailed eligibility criteria used for each stage of study selection are provided in Online Resource 1.

Records were limited by publication year to capture articles investigating CRF published in the last decade. Language was restricted to English because the review team was not able to translate articles published from other languages. Publication type was limited to original research journal articles to reduce the likelihood of including duplicate information published from secondary sources (e.g., review articles). At the full-text screening stage, articles were excluded in the order in which criteria are listed in Online Resource 1.

### Information sources and search strategy

A biomedical librarian (AAL) searched four electronic databases: PubMed [US National Library of Medicine], Embase [Elsevier], CINAHL Plus [EBSCOhost], PsycNet: PsycINFO and PsycARTICLES [American Psychological Association]. EndNote 20 (Clarivate Analytics, Philadelphia, Pennsylvania) was used to collect and manage records (e.g., remove duplicates) resulting from the search. The searches were conducted in January 2021 and limited to the English language for articles published from January 2010–December 2020. A combination of keywords and controlled vocabulary terms (e.g., CINAHL Subject Headings, EMTREE, MeSH, Thesaurus of Psychological Terms) was used for each concept of interest (e.g., cancer survivors, fatigue). Search strategies excluded animal studies and specific publication types (e.g., conference abstracts, editorials, reviews). See Online Resource 2 for the final search strategies and filters for each database.

### Study selection

A two-stage screening process was conducted in Covidence (Veritas Health Innovation, Melbourne, Australia): title and abstract screening and full-text screening. Before commencing, a pilot for each stage of screening was performed by three reviewers (KFK, TW, CB) to ensure a shared understanding and application of the eligibility criteria. Each pilot consisted of a random sample of records selected by the biomedical librarian: *n* = 68 for title and abstract screening and *n* = 15 for full-text screening. After completing each pilot, the review team met to discuss resulting discrepancies and modify the eligibility criteria accordingly.

The same three reviewers participated in both official screening stages, with two reviewers independently voting on each record (i.e., indicating pass/fail according to eligibility criteria). Voting discrepancies at both screening stages were discussed at weekly team meetings with the principal investigator (LS) and biomedical librarian to reach consensus and provide a deciding vote. A final review of all excluded articles at both screening stages was conducted by one reviewer (KFK) to ensure that articles were not erroneously excluded.

### Data charting process and data items

A data charting form (Online Resource 3) was developed in Covidence by one reviewer (KFK) with input and final approval from the review team. Three reviewers (KFK, TW, CB) piloted the data charting process using *n* = 5 articles, and they met with the review team afterward to discuss changes and finalize the data charting form. The same three reviewers participated in official data charting with data items from each article collected independently by two reviewers. Throughout the data charting process, questions were discussed with the review team on a weekly basis. Discrepancies in the collected data were reconciled by a single reviewer (KFK) who consulted with the principal investigator as needed.

The following data items were extracted for each aim:

- Aim 1 (Definitions): Definition Multidimensional (yes, no, not provided), NCCN’s Definition (yes, no)
- Aim 2 (Assessment): Measure Names, Measure Validation (yes, no), Measure Validation Description, Measure Timepoint (before, during, or after primary treatment), Multidimensional Measures (yes, no), Measure Dimensions, Outcomes Related to Dimensions
- Aim 3 (Sample and Article Information): Study Location (country), Study Design (longitudinal vs. cross-sectional, quantitative vs. qualitative, experimental vs. observational), Cancer Treatment, Cancer Intervention, Cancer Diagnosis, Sample Size (number of males, females, total sample), Sample Age (range if available; otherwise mean ± SD), Sample Race

We originally intended, and attempted, to extract CRF definitions and the dimensions contained therein, but the majority of articles did not include a clear definition for CRF (e.g., CRF is defined as…). Instead, many articles used various descriptions of CRF based on previous literature or patient report (e.g., described feelings of fatigue as…) that were scattered throughout the article’s introduction. Thus, it was extremely challenging to differentiate CRF definitions from descriptions, rendering it difficult to identify how CRF was operationalized for each study. As a result, we did not extract CRF definitions and their dimensions; instead, we focused on whether the definitions/descriptions were multidimensional (i.e., used the term multidimensional or listed multiple dimensions) and if they used the same definition as NCCN.

### Data synthesis

For data cleaning and synthesis, Microsoft Excel (Microsoft Corporation, Redmond, Washington) was used by three reviewers (KFK, JW, AAL). Descriptive statistics, a key characteristics table, and other tables and figures specific to the review’s aims are reported below.

## Results

### Selection of sources of evidence

**Fig. 1:**
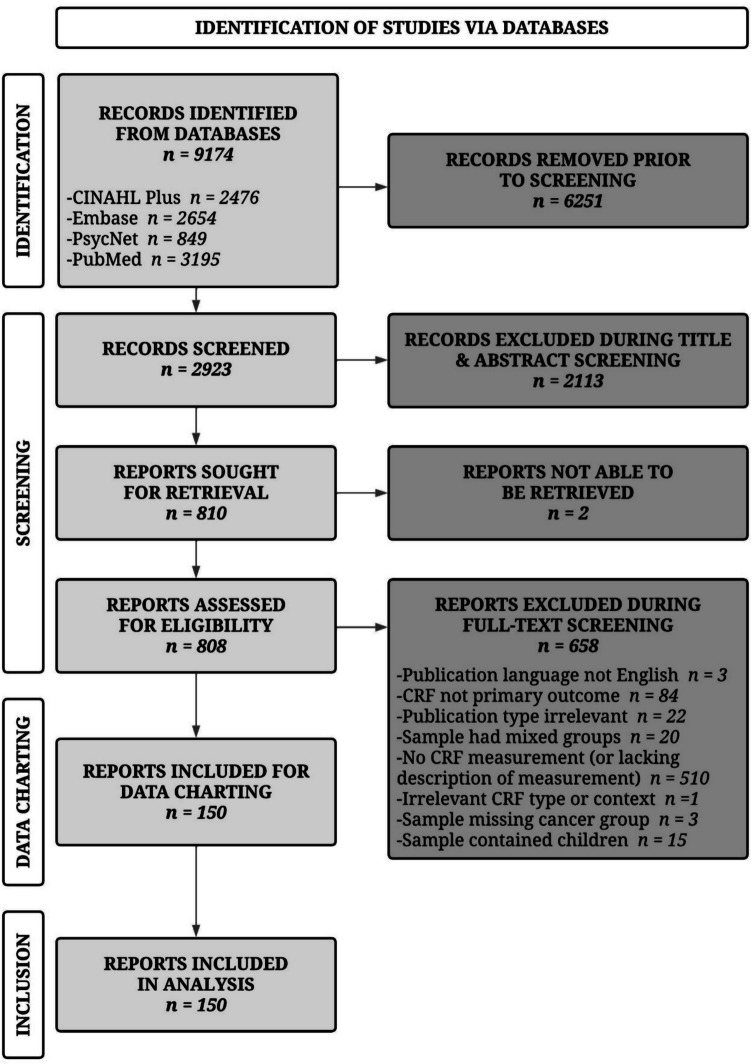
PRISMA Flow Diagram

The selection of evidence sources resulting from the search across four databases is provided in Fig. 1. The search yielded 9174 total records, of which 6251 duplicates were identified and removed, leaving 2923 unique records to be screened. After screening the titles and abstracts of these records, 2113 were excluded and 2 were unable to be retrieved, leaving 808 for full-text review. During full-text screening, 658 were excluded for reasons detailed in Fig. 1; the most common reason for exclusion was failing to measure (or describe the measurement of) CRF (*n* = 510). In total, 150 records were included in the final analysis.

After data extraction, the following data items were recoded into categories to summarize the data: cancer treatment, cancer intervention, race, cancer diagnosis, fatigue dimensions assessed (quantitative studies only). Original and recoded data for *all* extracted data items are provided by article in Online Resource 4 (CRF measure data items) and Online Resource 5 (all other data items).

### How CRF was defined

Data items relevant to CRF definitions are provided *by article* in Table 1 and Online Resource 5. Out of 150 total articles, 48 (32%) failed to provide a definition/description for CRF. Of the 102 articles (68%) that did provide CRF definitions/descriptions, 92 (90%) provided multidimensional definitions/descriptions and 42 (41%) used the NCCN CRF definition.

**Table 1:** Description of Key Characteristics by Article

Article Reference – Authors (year)	Study Location (Country)	Total Sample Size	Cancer Diagnoses within the Sample	Multi-Dimensional CRF Definition	Used NCCN's CRF Definition	Measure Name for CRF Assessment	Dimensions of CRF Assessment (Quantitative Study Design Only)
Abu Obead et al. (2014a)	Jordan	43	Blood Dyscrasias, Breast Cancer, GI Malignancies, GU Malignancies, Lung Cancer	Yes	No	Piper Fatigue Scale-Revised (PFS-R)	Affective, Behavioral, Cognitive, Sensory
Abu Obead et al. (2014b)	Jordan	43	Blood Dyscrasias, Breast Cancer, GI Malignancies, GU Malignancies, Lung Cancer	Yes	Yes	Piper Fatigue Scale-Revised (PFS-R)	Affective, Behavioral, Cognitive, Sensory
Akbas et al. (2021)	Turkey	50	GU Malignancies, Unspecified	Yes	No	Piper Fatigue Scale-Revised (PFS-R)	Affective, Behavioral, Cognitive, Sensory
Alfano et al. (2012)	United States	633	Breast Cancer	Not Reported	Not Reported	Piper Fatigue Scale-Revised (PFS-R)	Affective, Behavioral, Cognitive, Sensory
Ancoli-Israel et al. (2012)	United States	39	Breast Cancer	Yes	Yes	Multidimensional Fatigue Symptom Inventory-Short Form (MFSI-SF)	Emotional, Mental, Physical, Vigor
Andic et al. (2020)	United States	88	Breast Cancer	Yes	Yes	Multidimensional Fatigue Inventory (MFI‐20)	Mental, Motivational, Physical, Reduced Activity
Annunziata et al. (2013)	Italy	105	Unspecified	Yes	Yes	Piper Fatigue Scale-Revised (PFS-R)	Affective, Behavioral, Cognitive, Emotional, Sensory
Ansari et al. (2019)	Iran	71	Blood Dyscrasias, Unspecified	Yes	No	Multidimensional Fatigue Inventory (MFI‐20)	Mental, Motivational, Physical, Reduced Activity
Avelar et al. (2019)	Brazil	60	Head and Neck Cancer	Yes	No	Piper Fatigue Scale-Revised (PFS-R)	Affective, Behavioral, Sensory
Bang et al. (2016)	Korea	31	Breast Cancer, GI Malignancies, Lung Cancer	Yes	Yes	Other (Q Methodology)	Behavioral, Cognitive, Emotional, Physical, Other(1): Spiritual
Barton et al. (2013)	United States	341	Blood Dyscrasias, Breast Cancer, GI Malignancies, GU Malignancies	Not Reported	Not Reported	Multidimensional Fatigue Symptom Inventory-Short Form (MFSI-SF)	Affective, Emotional, Mental, Physical, Vigor
Björneklett et al. (2013)	Sweden	382	Breast Cancer	Not Reported	Not Reported	Chalder Fatigue Scale	Mental, Physical
Blaney et al. (2013)	Ireland	454	Blood Dyscrasias, Bone Cancer, Breast Cancer, CNS Malignancies, GI Malignancies, GU Malignancies, Head and Neck Cancer	Not Reported	Not Reported	Multidimensional Fatigue Symptom Inventory-Short Form (MFSI-SF)	Emotional, Mental, Physical, Vigor
Boing et al. (2018)	Brazil	122	Breast Cancer	Yes	No	Piper Fatigue Scale-Revised (PFS-R)	Affective, Behavioral, Sensory
Borneman et al. (2011)	United States	280	Breast Cancer, GI Malignancies, GU Malignancies, Lung Cancer	Not Reported	Not Reported	Piper Fatigue Scale-Revised (PFS-R)	Affective, Behavioral, Cognitive, Sensory
Bortolon et al. (2014)	France	60	Breast Cancer	Yes	Yes	Multidimensional Fatigue Inventory (MFI‐20)	Mental, Motivational, Physical
Bourmaud et al. (2017)	France	212	Blood Dyscrasias, Breast Cancer, GI Malignancies, GU Malignancies, Lung Cancer, Unspecified	Yes	Yes	Piper Fatigue Scale-Revised (PFS-R)	Affective, Behavioral, Cognitive, Sensory
Buffart et al. (2013)	Netherlands	119	Blood Dyscrasias, Breast Cancer, GI Malignancies, GU Malignancies, Unspecified	Not Reported	Not Reported	Multidimensional Fatigue Inventory (MFI‐20)	Mental, Motivational, Physical, Reduced Activity
Buffart et al. (2014)	Netherlands	209	Blood Dyscrasias, Breast Cancer, GI Malignancies, GU Malignancies, Lung Cancer, Unspecified	Not Reported	Not Reported	Multidimensional Fatigue Inventory (MFI‐20)	Mental, Motivational, Physical, Reduced Activity
Busson et al. (2019)	France	3642	Blood Dyscrasias	Not Reported	Not Reported	Multidimensional Fatigue Inventory (MFI‐20)	Mental, Motivational, Physical, Reduced Activity
Buyukbayram & Citlik Saritas (2021)	Turkey	180	Breast Cancer, GI Malignancies, Lung Cancer, Unspecified	Not Reported	Not Reported	Piper Fatigue Scale-Revised (PFS-R)	Affective, Behavioral, Cognitive, Sensory
Cantarero-Villaneuva et al. (2011)	Spain	67	Breast Cancer	No	No	Piper Fatigue Scale-Revised (PFS-R)	Affective, Behavioral, Cognitive, Sensory
Cantarero-Villanueva et al. (2012)	Spain	95	Breast Cancer	Not Reported	Not Reported	Piper Fatigue Scale-Revised (PFS-R)	Affective, Behavioral, Cognitive, Sensory
Cantarero-Villanueva et al. (2013a)	Spain	40	Breast Cancer	Not Reported	Not Reported	Piper Fatigue Scale-Revised (PFS-R)	Affective, Behavioral, Cognitive, Sensory
Cantarero-Villanueva et al. (2013b)	Spain	61	Breast Cancer	No	No	Piper Fatigue Scale-Revised (PFS-R)	Affective, Behavioral, Cognitive, Sensory
Carayol et al. (2019)	France	143	Breast Cancer	Yes	Yes	Multidimensional Fatigue Inventory (MFI‐20)	Mental, Motivational, Physical, Reduced Activity
Chae et al. (2018)	Singapore	108	Breast Cancer	Yes	Yes	Multidimensional Fatigue Symptom Inventory-Short Form (MFSI-SF)	Emotional, Mental, Physical, Vigor
Charalambous & Kouta (2016)	Cyprus	148	GU Malignancies	Yes	Yes	Cancer Fatigue Scale (CFS); Other (Interview)	Affective, Cognitive, Physical
Charalambous et al. (2016)	Cyprus	208	Breast Cancer, GU Malignancies	Not Reported	Not Reported	Cancer Fatigue Scale (CFS)	Affective, Cognitive, Physical
Chen, Ahmad, & Ismail (2019)	Malaysia	106	Blood Dyscrasias, Bone Cancer, Breast Cancer, CNS Malignancies, GI Malignancies, GU Malignancies, Head and Neck Cancer, Lung Cancer, Skin Cancer, Unspecified	Not Reported	Not Reported	Chalder Fatigue Scale	Mental, Physical, Other(1): Energy
Corbett et al. (2016)	Ireland	18	Blood Dyscrasias, Breast Cancer, GI Malignancies, GU Malignancies	Yes	Yes	Other (Focus Group)	Not Applicable
Cordero, Dimsdale, & Navarro (2015)	United States	35	Breast Cancer, GU Malignancies	Yes	Yes	Multidimensional Fatigue Symptom Inventory-Short Form (MFSI-SF); Other (Focus Group)	Emotional, Mental, Physical, Vigor
Crouch & Von Ah (2018)	United States	68	Breast Cancer	Yes	No	Attentional Function Index-13	Attention
Cuesta-Vargas et al. (2019)	Spain	43	Breast Cancer	Yes	No	Piper Fatigue Scale-Revised (PFS-R)	Affective, Behavioral, Cognitive, Sensory
Cuesta-Vargas et al. (2020)	Spain	70	Breast Cancer	Yes	No	Other (30-Second Sit-to-Stand)	Other(1): Physiologic Fatigue
de Raaf et al. (2012)	Netherlands	92	Breast Cancer, GI Malignancies, GU Malignancies, Unspecified	Yes	No	Multidimensional Fatigue Inventory (MFI‐20)	Mental, Physical
Dolgoy, O'Krafka, & McNeely (2020)	Canada	25	Head and Neck Cancer	Yes	No	Other (Written Questionnaire)	Not Applicable
Eyl et al. (2020)	Germany	1781	GI Malignancies	Not Reported	Not Reported	EORTC QLQ-C30; Fatigue Assessment Questionnaire (FAQ)	Affective, Cognitive, Physical
Feng, Espina, & Saligan (2018)	United States	36	GU Malignancies	Yes	Yes	Piper Fatigue Scale-Revised (PFS-R)	Cognitive
Fernández-de-las-Peñas et al. (2012)	Spain	128	Breast Cancer	Not Reported	Not Reported	Piper Fatigue Scale-Revised (PFS-R)	Affective, Behavioral, Cognitive, Sensory
Fox et al. (2020)	United States	128	Breast Cancer	Yes	Yes	Multidimensional Fatigue Symptom Inventory-Short Form (MFSI-SF)	Emotional, Mental, Physical, Vigor
Fu, Guang, & Gao (2018)	China	83	GU Malignancies	Not Reported	Not Reported	Chalder Fatigue Scale; Multidimensional Fatigue Inventory (MFI‐20)	Mental, Motivational, Physical, Reduced Activity
Gascón et al. (2013)	Spain	667	Blood Dyscrasias, Breast Cancer, GI Malignancies, GU Malignancies, Head and Neck Cancer, Lung Cancer, Unspecified	Not Reported	Not Reported	PERFORM Questionnaire	Other(4): Activities of Daily Living, Attitudes, Beliefs, Physical Limitations
George et al. (2013)	United States	710	Breast Cancer	Yes	No	Piper Fatigue Scale-Revised (PFS-R)	Affective, Behavioral, Cognitive, Sensory
George et al. (2014)	United States	770	Breast Cancer	Yes	No	Piper Fatigue Scale-Revised (PFS-R)	Affective, Behavioral, Cognitive, Sensory
Gjerset et al. (2017)	Norway	564	Blood Dyscrasias, Bone Cancer, Breast Cancer, CNS Malignancies, GI Malignancies, GU Malignancies, Head and Neck Cancer, Lung Cancer, Neuroendocrine Tumors, Unspecified	Yes	Yes	Chalder Fatigue Scale	Mental, Physical
Grégoire et al. (2020)	Belgium	95	Blood Dyscrasias, Breast Cancer, CNS Malignancies, GI Malignancies, GU Malignancies, Head and Neck Cancer, Lung Cancer, Skin Cancer	Not Reported	Not Reported	Multidimensional Fatigue Inventory (MFI‐20)	Mental, Motivational, Physical, Reduced Activity
Haas (2011)	United States	73	Breast Cancer	No	No	Piper Fatigue Scale-Revised (PFS-R)	Affective, Behavioral, Cognitive, Sensory
Hampson et al. (2015)	United States	23	Breast Cancer	Not Reported	Not Reported	Multidimensional Fatigue Inventory (MFI‐20)	Mental, Motivational, Physical, Reduced Activity
Hayama & Inoue (2012)	Japan	23	GI Malignancies, GU Malignancies	Yes	Yes	Cancer Fatigue Scale (CFS)	Affective, Cognitive, Physical
Horsboel et al. (2015)	Denmark	196	Blood Dyscrasias, Unspecified	Not Reported	Not Reported	Multidimensional Fatigue Inventory (MFI‐20)	Mental, Motivational, Physical, Reduced Activity
Huang et al. (2021)	China	427	Breast Cancer	Yes	Yes	Chinese Cancer-Related Fatigue Assessment Scale	Emotional, Mental, Physical
Ikeuchi et al. (2020)	Japan	249	Breast Cancer	Yes	Yes	Cancer Fatigue Scale (CFS)	Affective, Cognitive, Physical
Inglis et al. (2020)	United States	565	Breast Cancer	No	No	Multidimensional Fatigue Symptom Inventory-Short Form (MFSI-SF)	Emotional, Mental, Physical, Vigor
Inglis et al. (2021)	United States	105	Breast Cancer	Yes	Yes	Multidimensional Fatigue Symptom Inventory-Short Form (MFSI-SF)	Emotional, Mental, Physical, Vigor
Jain et al. (2012)	United States	76	Breast Cancer	Not Reported	Not Reported	Multidimensional Fatigue Symptom Inventory-Short Form (MFSI-SF)	Emotional, Mental, Physical, Vigor
James et al. (2015)	United Kingdom	176	Breast Cancer, CNS Malignancies, GI Malignancies, GU Malignancies, Head and Neck Cancer, Lung Cancer, Skin Cancer	Yes	No	EORTC QLQ-FA13	Cognitive, Emotional, Physical
Jang et al. (2022)	Korea	229	Breast Cancer	Not Reported	Not Reported	Piper Fatigue Scale-Revised (PFS-R)	Affective, Behavioral, Cognitive, Sensory
Johnson et al. (2018)	Canada	81	Breast Cancer, GI Malignancies, GU Malignancies, Lung Cancer, Unspecified	Yes	Yes	Multidimensional Fatigue Symptom Inventory-Short Form (MFSI-SF)	Emotional, Mental, Physical, Vigor
Jong et al. (2018)	Netherlands	83	Breast Cancer	Yes	Yes	Fatigue Quality List (FQL); Multidimensional Fatigue Inventory (MFI‐20)	Mental, Motivational, Physical, Reduced Activity
Kalter et al. (2016)	Netherlands	277	Blood Dyscrasias, Breast Cancer, GI Malignancies, GU Malignancies	Not Reported	Not Reported	Multidimensional Fatigue Inventory (MFI‐20)	Physical
Kampshoff et al. (2015)	Netherlands	277	Blood Dyscrasias, Breast Cancer, GI Malignancies, GU Malignancies	Not Reported	Not Reported	Multidimensional Fatigue Inventory (MFI‐20)	Mental, Motivational, Physical, Reduced Activity
Kampshoff, van Dongen et al. (2018)	Netherlands	277	Blood Dyscrasias, Breast Cancer, GI Malignancies, GU Malignancies	Not Reported	Not Reported	Multidimensional Fatigue Inventory (MFI‐20)	Mental, Motivational, Physical, Reduced Activity
Kim (Sue) et al. (2020a)	Korea	48	Breast Cancer	Yes	Yes	Piper Fatigue Scale-Revised (PFS-R)	Affective, Behavioral, Cognitive, Sensory
Kim (Sue) et al. (2020b)	Korea	16	Breast Cancer	Yes	Yes	Other (Focus Group)	Not Applicable
Kim (Sung Hae) et al. (2020)	Korea	40	Breast Cancer	Yes	No	Piper Fatigue Scale-Revised (PFS-R); Other (Enzyme-Linked Immunosorbent Assay Kits)	Affective, Behavioral, Cognitive, Sensory, Other(1): Physiologic Fatigue
Köhler et al. (2014)	Germany	329	GU Malignancies	Yes	Yes	Multidimensional Fatigue Inventory (MFI‐20)	Mental, Motivational, Physical, Reduced Activity
Koole et al. (2020)	Netherlands	*325	GI Malignancies	Yes	No	Checklist Individual Strength (CIS)	Motivational, Physical, Other(2): Concentration Problems, Subjective Feelings Of Fatigue
Kröz et al. (2017)	Germany	84	Breast Cancer	Yes	Yes	Cancer Fatigue Scale (CFS)	Affective, Cognitive, Physical
Kühl et al. (2018)	Germany	1389	Breast Cancer	Yes	No	Fatigue Assessment Questionnaire (FAQ)	Affective, Cognitive, Physical
Kyrdalen et al. (2010a)	Norway	521	GU Malignancies	Yes	No	Chalder Fatigue Scale	Mental, Physical
Kyrdalen et al. (2010b)	Norway	423	GU Malignancies	Not Reported	Not Reported	Chalder Fatigue Scale	Mental, Physical
Lacourt et al. (2018)	United States	47	Blood Dyscrasias, Breast Cancer, Head and Neck Cancer, Unspecified	Yes	No	Checklist Individual Strength (CIS); Multidimensional Fatigue Symptom Inventory-Short Form (MFSI-SF); Other (Visual Analogue Scale)	Motivational, Physical, Other(1): Momentary
Lee et al. (2020)	France	2799	Breast Cancer	Yes	No	EORTC QLQ-FA12	Cognitive, Emotional, Physical
Levkovich, Cohen, & Karkabi (2019)	Israel	13	Breast Cancer	No	No	Other (Interview)	Not Applicable
Li & Yuan (2011)	China	252	Breast Cancer	Not Reported	Not Reported	Piper Fatigue Scale-Revised (PFS-R)	Affective, Cognitive, Emotional, Mental, Physical, Sensory, Other(1): Severity
Lin et al. (2019)	United States	358	Blood Dyscrasias, Breast Cancer, GI Malignancies, GU Malignancies, Unspecified	Yes	No	Multidimensional Fatigue Symptom Inventory-Short Form (MFSI-SF)	Emotional, Mental, Physical, Vigor
Lin et al. (2021)	China	100	Lung Cancer	Yes	No	Cancer Fatigue Scale (CFS)	Affective, Cognitive, Physical
Liu et al. (2012)	United States	97	Breast Cancer	Yes	Yes	Multidimensional Fatigue Symptom Inventory-Short Form (MFSI-SF)	Emotional, Mental, Physical, Vigor
Liu et al. (2020)	China	133	Breast Cancer	Yes	No	Piper Fatigue Scale-Revised (PFS-R)	Affective, Behavioral, Cognitive, Sensory
Mardanian-Dehkordi & Kahangi (2018)	Iran	119	Unspecified	Yes	No	Cancer Fatigue Scale (CFS)	Cognitive, Emotional, Physical
Matsugaki et al. (2018)	Japan	18	Blood Dyscrasias, Unspecified	Yes	Yes	Cancer Fatigue Scale (CFS)	Affective, Cognitive, Physical
Miaskowski et al. (2011)	United States	185	Breast Cancer, CNS Malignancies, GU Malignancies, Lung Cancer	Yes	No	Attentional Function Index-16; Visual Analogue Scale for Fatigue (VAS-F)	Attention, Physical, Other(1): Energy
Mijwel et al. (2019)	Sweden	206	Breast Cancer	Yes	No	Piper Fatigue Scale-Revised (PFS-R)	Affective, Behavioral, Cognitive, Emotional, Physical, Sensory
Miura, Ando, & Imai (2016)	Japan	93	Breast Cancer	Yes	No	Cancer Fatigue Scale (CFS)	Affective, Attention, Cognitive, Physical, Other(1): Memory
Molassiotis et al. (2012)	United Kingdom	302	Breast Cancer	Not Reported	Not Reported	Multidimensional Fatigue Inventory (MFI‐20)	Mental, Motivational, Physical, Reduced Activity
Molassiotis et al. (2013)	United Kingdom	197	Breast Cancer	Not Reported	Not Reported	Multidimensional Fatigue Inventory (MFI‐20)	Mental, Motivational, Physical, Reduced Activity
Mulhaeriah et al. (2018)	Indonesia	42	GU Malignancies	Yes	No	Piper Fatigue Scale-Revised (PFS-R)	Affective, Behavioral, Cognitive
Nilsson et al. (2020)	Norway	240	Blood Dyscrasias, Breast Cancer, GI Malignancies, GU Malignancies	Yes	No	Chalder Fatigue Scale	Mental, Physical
Nowe et al. (2019)	Germany	577	Blood Dyscrasias, Bone Cancer, Breast Cancer, GI Malignancies, GU Malignancies, Head and Neck Cancer, Skin Cancer, Unspecified	Yes	Yes	EORTC QLQ-FA12	Cognitive, Emotional, Physical
Oei et al. (2020)	Germany	319	Breast Cancer	Yes	No	Cancer Fatigue Scale (CFS)	Affective, Cognitive, Physical
Oldervoll et al. (2011)	Norway	231	Blood Dyscrasias, Breast Cancer, GI Malignancies, GU Malignancies, Lung Cancer, Unspecified	Not Reported	Not Reported	Chalder Fatigue Scale	Mental, Physical
Otaghi et al. (2019)	Iran	95	Blood Dyscrasias, Breast Cancer, GI Malignancies, Unspecified	Yes	Yes	Multidimensional Fatigue Inventory (MFI‐20)	Mental, Motivational, Physical, Reduced Activity
Parveen et al. (2019)	India	71	Bone Cancer, Breast Cancer, GI Malignancies, Head and Neck Cancer, Lung Cancer	Yes	Yes	Multidimensional Fatigue Inventory (MFI‐20)	Mental, Motivational, Physical, Reduced Activity
Penner et al. (2020)	United States	13	Breast Cancer	Yes	Yes	Other (Interview)	Not Applicable
Pongthavornkamol et al. (2012)	Canada, Thailand	10	GI Malignancies, Lung Cancer	Yes	Yes	Other (Interview)	Not Applicable
Poort et al. (2021)	United States	23	GU Malignancies	Yes	No	Other (Interview)	Not Applicable
Regan & Hegarty (2017)	Ireland	362	Blood Dyscrasias, Breast Cancer, GI Malignancies	Yes	No	Piper Fatigue Scale-Revised (PFS-R); Other (Written Questionnaire)	Affective, Behavioral, Cognitive, Sensory
Reif et al. (2013)	Germany	234	Blood Dyscrasias, Breast Cancer, GI Malignancies, GU Malignancies	Yes	Yes	Fatigue Assessment Questionnaire (FAQ)	Affective, Cognitive, Physical
Rissanen et al. (2014)	Sweden	155	Breast Cancer	Yes	No	Multidimensional Fatigue Inventory (MFI‐20)	Mental, Motivational, Physical, Reduced Activity
Rozmus et al. (2021)	Not Reported	*28	Blood Dyscrasias, Breast Cancer, GI Malignancies, GU Malignancies	Yes	No	Other (Online Discussion Forum)	Not Applicable
Sadeghi, Gozali, & Moghaddam Tabrizi (2016)	Iran	135	Breast Cancer	No	No	Cancer Fatigue Scale (CFS)	Affective, Cognitive, Physical
Salmon et al. (2017)	France	466	Breast Cancer	Yes	No	Multidimensional Fatigue Inventory (MFI‐20)	Mental, Motivational, Physical, Reduced Activity
Sato (2020)	Japan	10	Breast Cancer, GI Malignancies, GU Malignancies, Lung Cancer	Not Reported	Not Reported	Other (Written Questionnaire)	Mental, Physical, Other(1): Comprehensive Fatigue
Schad et al. (2020)	Germany	231	Breast Cancer	Not Reported	Not Reported	Cancer Fatigue Scale (CFS)	Affective, Cognitive, Physical
Schjolberg et al. (2014)	Norway	160	Breast Cancer	No	No	Chalder Fatigue Scale	Mental, Physical
Schlemmer et al. (2015)	Germany	100	Bone Cancer, Breast Cancer, GI Malignancies, GU Malignancies, Head and Neck Cancer, Lung Cancer	No	No	Cancer Fatigue Scale (CFS)	Affective, Cognitive, Physical
Schmidt et al. (2015a)	Germany	1928	Breast Cancer	Yes	No	Fatigue Assessment Questionnaire (FAQ)	Affective, Cognitive, Physical
Schmidt et al. (2015b)	Germany	95	Breast Cancer	Not Reported	Not Reported	Fatigue Assessment Questionnaire (FAQ)	Affective, Cognitive, Physical
Schmidt et al. (2016a)	Germany	265	Breast Cancer	Yes	No	Fatigue Assessment Questionnaire (FAQ)	Affective, Cognitive, Physical
Schmidt et al. (2016b)	Germany	103	Breast Cancer	Yes	No	Fatigue Assessment Questionnaire (FAQ)	Affective, Cognitive, Physical
Schmidt et al. (2018)	Germany	255	Breast Cancer	Yes	Yes	Fatigue Assessment Questionnaire (FAQ)	Affective, Cognitive, Physical
Schmitt et al. (2016)	Germany	26	Blood Dyscrasias, Breast Cancer, GI Malignancies, GU Malignancies, Unspecified	Not Reported	Not Reported	Multidimensional Fatigue Inventory (MFI‐20)	Mental, Motivational, Physical, Reduced Activity
Schreier et al. (2019)	United States	40	Breast Cancer	Not Reported	Not Reported	Piper Fatigue Scale-12 (PFS-12)	Affective, Behavioral, Cognitive, Sensory
Schulz et al. (2017)	Germany	203	Blood Dyscrasias, Breast Cancer, GI Malignancies, GU Malignancies, Lung Cancer	Yes	No	Multidimensional Fatigue Inventory (MFI‐20)	Mental, Motivational, Physical, Reduced Activity
Sha et al. (2015)	China	100	Lung Cancer	Yes	No	Cancer Fatigue Scale (CFS)	Affective, Cognitive, Physical
Shi et al. (2020)	Canada	147	Breast Cancer	No	No	Multidimensional Fatigue Inventory (MFI‐20)	Mental, Motivational, Physical, Reduced Activity
Singer et al. (2011)	Germany	1494	Blood Dyscrasias, Bone Cancer, Breast Cancer, CNS Malignancies, GI Malignancies, GU Malignancies, Head and Neck Cancer, Lung Cancer, Skin Cancer, Unspecified	Yes	No	Multidimensional Fatigue Inventory (MFI‐20)	Mental, Motivational, Physical, Reduced Activity
Sobel-Fox et al. (2013)	United States	52	Breast Cancer, GI Malignancies, GU Malignancies, Lung Cancer, Unspecified	Yes	Yes	Multidimensional Fatigue Symptom Inventory-Short Form (MFSI-SF)	Emotional, Mental, Physical, Vigor
Song et al. (2021)	China	80	GI Malignancies	Not Reported	Not Reported	Piper Fatigue Scale-Revised (PFS-R)	Behavioral, Cognitive, Emotional, Other(1): Perception
Sørensen et al. (2020)	Norway	160	Breast Cancer	No	No	Chalder Fatigue Scale	Mental, Physical
Spahn et al. (2013)	Germany	55	Breast Cancer	Yes	No	Multidimensional Fatigue Inventory (MFI‐20)	Mental, Motivational, Physical, Reduced Activity
Spichiger et al. (2012)	Switzerland	19	Blood Dyscrasias, Breast Cancer, GI Malignancies, Lung Cancer	Yes	No	Other (Interview)	Not Applicable
Sprod et al. (2010)	United States	114	Breast Cancer	Not Reported	Not Reported	Piper Fatigue Scale-Revised (PFS-R); Other (Muscular Test Battery)	Affective, Behavioral, Cognitive, Emotional, Mental, Physical, Sensory, Other(1): Volitional
Sprod et al. (2015)	United States	97	Breast Cancer, Unspecified	Not Reported	Not Reported	Multidimensional Fatigue Symptom Inventory-Short Form (MFSI-SF)	Emotional, Mental, Physical, Vigor
Steindorf et al. (2014)	Germany	155	Breast Cancer	Not Reported	Not Reported	Fatigue Assessment Questionnaire (FAQ)	Affective, Cognitive, Physical
Steindorf et al. (2019)	Germany	47	GI Malignancies	Not Reported	Not Reported	Multidimensional Fatigue Inventory (MFI‐20)	Mental, Motivational, Physical, Reduced Activity
Strebkova (2020)	Bulgaria	66	Breast Cancer, GI Malignancies, GU Malignancies, Unspecified	Yes	Yes	Multidimensional Fatigue Inventory (MFI‐20)	Mental, Motivational, Physical, Reduced Activity
Sundbom et al. (2020)	Sweden	195	GI Malignancies	Not Reported	Not Reported	Multidimensional Fatigue Inventory (MFI‐20); Other (Timed Stand Test)	Mental, Motivational, Physical, Reduced Activity
Susanne et al. (2019)	Germany	948	Blood Dyscrasias, Breast Cancer, GI Malignancies, GU Malignancies, Head and Neck Cancer, Skin Cancer, Unspecified	Yes	Yes	EORTC QLQ-FA12	Cognitive, Emotional, Physical
Tabrizi & Alizadeh (2017)	Iran	150	Breast Cancer	Yes	No	Cancer Fatigue Scale (CFS)	Affective, Cognitive, Physical
Thong et al. (2018)	Netherlands	1183	GI Malignancies	Yes	Yes	Multidimensional Fatigue Inventory (MFI‐20)	Mental, Motivational, Physical, Reduced Activity
Toh et al. (2019)	Singapore	136	Breast Cancer	Yes	Yes	Multidimensional Fatigue Symptom Inventory-Short Form (MFSI-SF)	Emotional, Mental, Physical, Vigor
Toh et al. (2020)	Singapore	155	Breast Cancer	Yes	Yes	Multidimensional Fatigue Symptom Inventory-Short Form (MFSI-SF)	Emotional, Mental, Physical, Vigor
Travier et al. (2015)	Netherlands	164	Breast Cancer	Not Reported	Not Reported	Fatigue Quality List (FQL); Multidimensional Fatigue Inventory (MFI‐20)	Mental, Motivational, Physical, Reduced Activity, Other(4): Exhausting, Frightening, Frustrating, Pleasant
Tsai et al. (2010)	Taiwan	15	Breast Cancer	Yes	Yes	Other (Interview)	Not Applicable
Van Onselen et al. (2012)	United States	398	Breast Cancer	Not Reported	Not Reported	Attentional Function Index-16; Visual Analogue Scale for Fatigue (VAS-F)	Attention, Physical
Van Vulpen et al. (2016)	Netherlands	33	GI Malignancies	Yes	No	Fatigue Quality List (FQL); Multidimensional Fatigue Inventory (MFI‐20)	Mental, Motivational, Physical, Reduced Activity, Other(4): Exhausting, Frightening, Frustrating, Pleasant
van Vulpen et al. (2018)	Germany	130	Breast Cancer	Yes	No	Fatigue Assessment Questionnaire (FAQ); Multidimensional Fatigue Inventory (MFI‐20)	Mental, Physical
van Waart et al. (2015)	Netherlands	230	Breast Cancer	Not Reported	Not Reported	Multidimensional Fatigue Inventory (MFI‐20)	Mental, Motivational, Physical, Reduced Activity
Von Ah et al. (2017)	United States	68	Breast Cancer	Yes	No	Attentional Function Index-13	Attention, Mental
Wang et al. (2013)	China	300	Breast Cancer	Not Reported	Not Reported	Cancer Fatigue Scale (CFS)	Affective, Cognitive, Physical
Watson & van Kessel (2018)	New Zealand	15	Blood Dyscrasias, Bone Cancer, Breast Cancer, GI Malignancies, GU Malignancies, Smooth Muscle Tumor	Yes	No	Other (Online Blog)	Not Applicable
Wei & Li (2018)	China	70	GI Malignancies	Yes	Yes	Cancer Fatigue Scale (CFS)	Affective, Cognitive, Physical
Weißflog et al. (2015)	Germany	106	Breast Cancer	Yes	No	Multidimensional Fatigue Inventory (MFI‐20)	Mental, Motivational, Physical, Reduced Activity
Witlox et al. (2018)	Netherlands	128	Breast Cancer, GI Malignancies	Not Reported	Not Reported	Multidimensional Fatigue Inventory (MFI‐20)	Mental, Motivational, Physical, Reduced Activity
Xie et al. (2020)	China	86	GI Malignancies	Not Reported	Not Reported	Fatigue Assessment Questionnaire (FAQ)	Affective, Cognitive, Physical
Zetzl et al. (2021)	Germany	159	Blood Dyscrasias, Breast Cancer, CNS Malignancies, GI Malignancies, GU Malignancies, Head and Neck Cancer, Lung Cancer, Skin Cancer, Unspecified	Yes	No	EORTC QLQ-FA12	Cognitive, Emotional, Physical
Zhang et al. (2017)	China	48	Breast Cancer	Not Reported	Not Reported	Multidimensional Fatigue Inventory (MFI‐20)	Mental, Motivational, Physical, Reduced Activity
Zhou et al. (2020)	China	73	GU Malignancies	Yes	No	Piper Fatigue Scale-Revised (PFS-R)	Behavioral, Cognitive, Emotional, Sensory

### How CRF was assessed

**Fig. 2:**
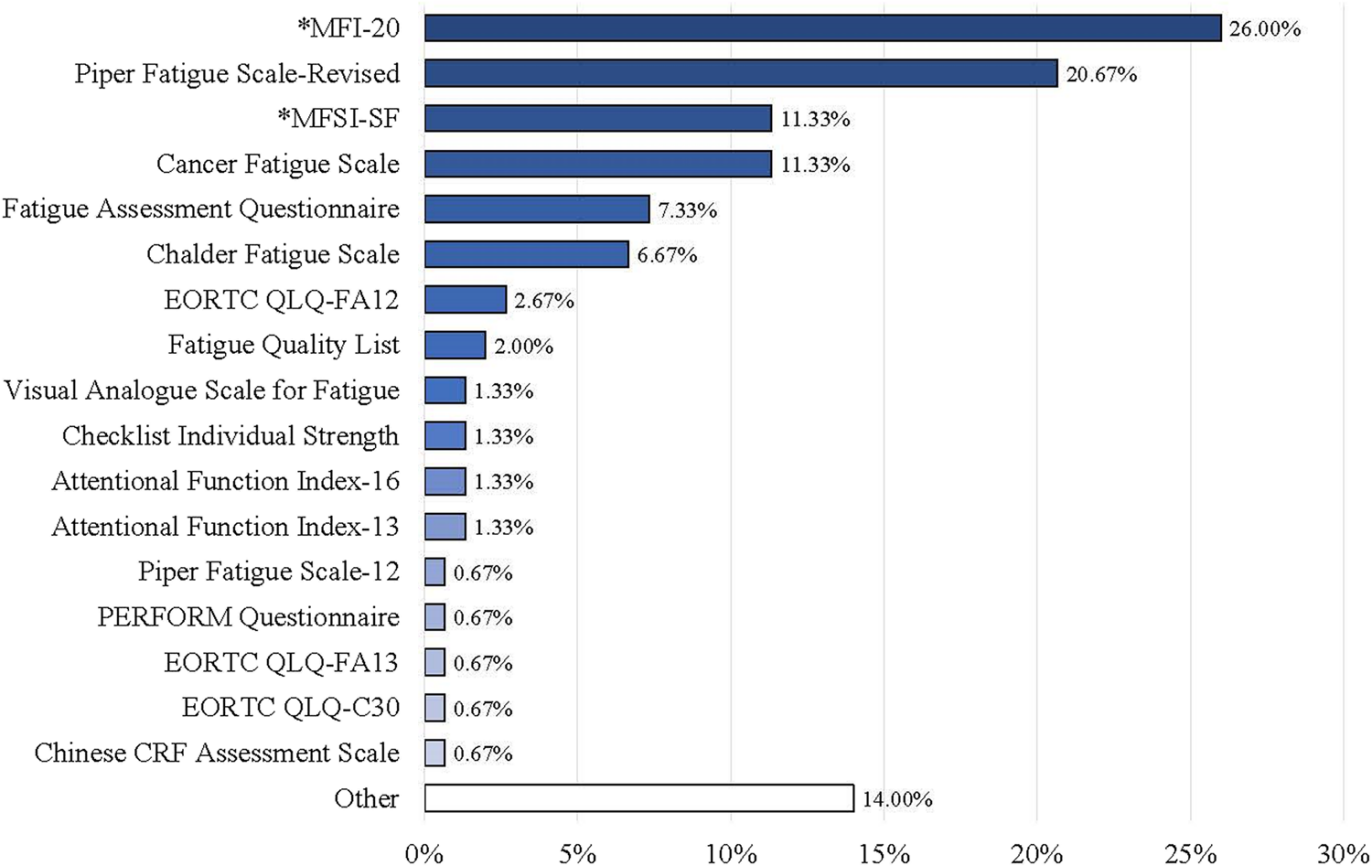
Percentage of All Articles (*n* = 150) by Cancer-Related Fatigue Measure. *Indicates Abbreviations: MFI-20 = Multidimensional Fatigue Inventory-20 Item Version; MFSI-SF = Multidimensional Fatigue Symptom Inventory-Short Form Version. Note: The “Other” category consists of 21 articles that used fatigue assessments that were not given a specific name, and contains the following subgroups: interview *n* = 7, focus group *n* = 3, written survey *n* = 3, enzyme-linked immunosorbent assay (ELISA) kit *n* = 1, muscular test battery *n* = 1, online blog *n* = 1, online discussion forum *n* = 1, Q methodology *n* = 1, 30-s sit-to-stand test *n* = 1, timed stand test *n* = 1, visual analogue scale (source uncited) *n* = 1

**Fig. 3:**
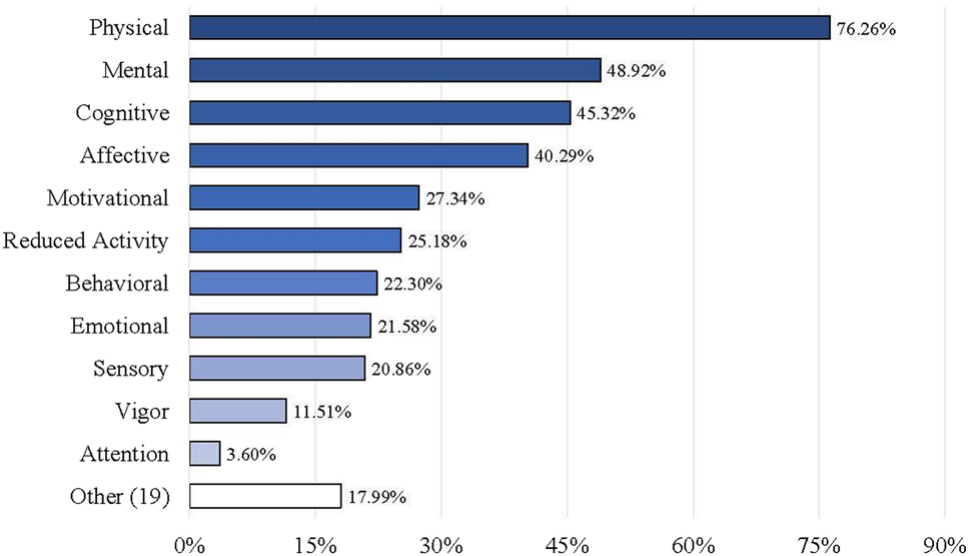
Percentage of Articles with Quantitative Designs (*n* = 139) for Each Fatigue Dimension Assessed. Note: the “Other” category consists of fatigue dimensions that were found in two or fewer articles, including: activities of daily living *n* = 1, attitudes *n* = 1, beliefs *n* = 2, comprehensive fatigue *n* = 1, concentration problems *n* = 1, energy *n* = 1, exhausting *n* = 2, frightening *n* = 2, frustrating *n* = 2, memory *n* = 1, momentary *n* = 1, perception *n* = 1, physical limitations *n* = 1, physiologic fatigue *n* = 2, pleasant *n* = 2, severity *n* = 1, spiritual *n* = 1, subjective feelings of fatigue *n* = 1, volitional *n* = 1

The proportion of articles that used multidimensional CRF measures was 97% (quantitative studies only; *n* = 139) (see Online Resource 5). For articles that used the NCCN definition of CRF (*n* = 42), the most common assessments used were the Multidimensional Fatigue Symptom Inventory-Short Form (MFSI-SF; *n* = 10) and the Multidimensional Fatigue Inventory (MFI‐20; *n* = 9). Across *all* articles, 28 unique CRF measures were used, with 17 assessments consisting of known self-report measures and 11 general assessments lacking specific names (e.g., interviews, focus groups, online blogs) (Table 2). CRF measure names are provided *by article* in Table 1 and Online Resource 4 and *summarized* in Fig. 2, with the MFI-20 found to be the most common CRF measure (26%) used across all articles. Additional details (i.e., item number, scale format, dimensions assessed, scoring interpretation, reference period) regarding the most common CRF measures are *summarized* in Table 2, with measures most often consisting of 20 items (ranging from 11–30), Likert scale format, and present day as the reference period for CRF. In addition, the “Physical” dimension was the most common fatigue-related dimension included across CRF measures; however, even though a study may have included a certain measure to assess CRF, the authors may not have used all dimensions included in the measure, so we only extracted dimensions that were used. For example, if a study included the MFI-20 (which contains five dimensions) but the authors only assessed the dimension of physical fatigue, we would extract physical fatigue alone. A total of 30 unique dimensions resulted across articles (Table 1), with “Physical” (76%), “Mental” (49%), and “Cognitive” (45%) found to be the most common CRF dimensions (Fig. 3). Of the 166 total (non-unique) measures used across articles, only 91 (55%) included information on whether the measure was validated (Online Resource 4).

**Table 2:** Summary Information for the Most Common Clinical Measures Used to Assess Cancer-Related Fatigue

Measure Name & Hyperlinked Original Source	Total Item # & Scale Format	Measure Dimensions (Item #) *Non-Fatigue Dimensions	Higher Scores Indicate	Reference Period	# (%) Articles Using Measure
Attentional Function Index-16 Cimprich (1990)	16 Items *Visual Analogue*	Attentional Difficulties (4) Executive Functioning (12)	Less Fatigue	Present	2 (1.33)
Attentional Function Index-13 Cimprich, Visovatti, & Ronis (2011)	13 Items *Visual Analogue*	Attentional Lapses (3) Effective Action (7) Interpersonal Effectiveness (3)	Less Fatigue	Present	2 (1.33)
Cancer Fatigue Scale Okuyama et al. (2000)	15 Items *Likert (5-point)*	Affective (4) Cognitive (4) Physical (7)	More Fatigue	Present	17 (11.33)
Chalder Fatigue Scale Chalder et al. (1993)	11 Items *Bimodal OR* *Likert (4-point)*	Mental (4) Physical (7)	More Fatigue	Not Reported	10 (6.67)
Checklist Individual Strength Vercoulen et al. (1994)	20 Items *Likert (7-point)*	Activity (3) Concentration (5) Motivation (4) Subjective Fatigue (8)	More Fatigue	Past 2 Weeks	2 (1.33)
Chinese Cancer-Related Fatigue Assessment Scale Li & Yuan (2009)	20 Items *Likert (5-point)*	Emotional (7) Mental (5) Physical (8)	Not Reported	Not Reported	1 (0.67)
EORTC QLQ-C30 Aaronson et al. (1993)	30 Items *Bimodal* *Likert (4-point)* *Likert (7-point)*	Functioning Scales: *Cognitive (2) *Emotional (4) *Global Health *Physical (5) Quality of Life (2) *Role (2) *Social (2) Symptom Scales: Fatigue (3) *Appetite Loss (1) *Constipation (1) *Diarrhea (1) *Dyspnea (1) *Financial Impact (1) *Nausea and Vomiting (2) *Pain (2) *Sleep Disturbance (1)	More Fatigue	Past Week	1 (0.67)
EORTC QLQ-FA12 Weis et al. (2017)	12 Items *Likert (4-point)*	Cognitive (2) Emotional (3) *Interference (2) Physical (5)	More Fatigue	Past Week	4 (2.67)
EORTC QLQ-FA13 Weis et al. (2013)	13 Items *Likert (4-point)*	Cognitive (3) Emotional (4) *Interference (2) Physical (4)	More Fatigue	Past Week	1 (0.67)
Fatigue Assessment Questionnaire Glaus & Müller (2001)	20 Items *Likert (4-point)*	Affective (5) Cognitive (3) Physical (11)	More Fatigue	Not Reported	11 (7.33)
Fatigue Quality List Gielissen et al. (2007)	18 Items *Multiple Selection List*	Exhausting (4) Frightening (4) Frustrating (5) Pleasant (5)	More Fatigue	Past 2 Weeks	3 (2.00)
Multidimensional Fatigue Inventory (MFI-20) Smets et al. (1995) Smets et al. (1996)	20 Items *Likert (7-point)*	General (4) Mental (4) Physical (4) Reduced Activity (4) Reduced Motivation (4)	More Fatigue	Previous Days	39 (26.00)
	20 Items *Likert (5-point)*	General (4) Mental (4) Physical (4) Reduced Activity (4) Reduced Motivation (4)	More Fatigue	Not Reported	
Multidimensional Fatigue Symptom Inventory-Short Form (MFSI-SF) Stein et al. (1998) Stein et al. (2004)	30 Items *Likert (5-point)*	Emotional (6) General (6) Mental (6) Physical (6) Vigor (6)	More Fatigue	Past Week	17 (11.33)
PERFORM Questionnaire Baro et al. (2009)	12 Items *Likert (5-point)*	Activities of Daily Living (4) Beliefs and Attitudes (4) Physical Limitations (4)	More Fatigue	Past 2 Weeks	1 (0.67)
Piper Fatigue Scale-12 Reeve et al. (2012)	12 Items *Likert (11-point)*	Affective (3) Behavioral (3) Cognition/Mood (3) Sensory (3)	More Fatigue	Past 4 Weeks	1 (0.67)
Piper Fatigue Scale-Revised Piper et al. (1998)	27 Items *Likert (11-point)* *Open-Ended*	Affective (5) Behavioral/Severity (6) Cognition/Mood (6) *Unspecified (5) Sensory (5)	More Fatigue	Present	31 (20.67)
Visual Analogue Scale for Fatigue Lee, Hicks, & Nino-Murcia (1991)	18 Items *Visual Analogue*	Energy (5) Fatigue (13)	More Fatigue	Present	2 (1.33)

Due to the inconsistency with which articles reported the language versions of their respective fatigue measures, we coded all language versions of a measure in the same group with the original measure and only provided information on the original version. Due to this, it's important to note that translated versions may vary in item number and scoring from what is listed in this table. In addition, the number (percent) of articles that use each measure is indicated in the last column and is calculated out of 150 total articles (note that some articles used multiple measures, accounting for the total exceeding 100%; see Online Resource 1 for specific measure information by article). All measures reported in this table are self-report. An additional 21 articles (14.00%) used fatigue assessments that were classified as “Other” because they were not given a specific name. The “Other” category contains the following subgroups: interview *n=*7, focus group *n=*3, written survey *n=*3, enzyme-linked immunosorbent assay (ELISA) kit *n=*1, muscular test battery *n=*1, online blog *n=*1, online discussion forum *n=*1, Q methodology *n=*1, 30-second sit-to-stand test *n=*1, timed stand test *n=*1, visual analogue scale (source uncited) *n=*1

### Characteristics of included studies

Demographic and study design data items are provided *by article* in Table 1 and Online Resource 5, as well as *summarized* in Table 3. CRF has been studied across 31 countries, with the United States (19%) and Germany (16%) as the leading study locations. Sample sizes ranged from 10 to 3642 people, with samples of at least 100 (57%) occurring most frequently. Sample age (range if available; otherwise mean ± SD) and gender (number of females and/or males) were missing in 31% and 13% of articles, respectively. Of the 71 articles that reported age range, 83% included young adults (ages 18–39 years), 99% middle-aged adults (ages 40–64 years), and 93% older adults (ages 65 +); 76% of articles included all three age groups (young, middle, older). Race was only reported in 18% of articles; of the 27 articles that reported race, White was the most common subcategory (85%), followed by Other/Unknown (70%), African American or Black (48%), Asian (41%), American Indian or Alaska Native (15%), Native Hawaiian or Other Pacific Islander (4%). Breast cancer was by far the most common cancer diagnosis across articles (79%), with gastrointestinal and genitourinary malignancies trailing behind (35% and 33%, respectively). Chemotherapy (80%) and surgery (55%) were the most common cancer treatments (observational articles only; *n* = 86). Integrative medicine (54%) and physical exercise (41%) were the most prevalent CRF interventions (experimental articles only; *n* = 64). The majority of study designs were longitudinal (67%) vs. cross-sectional, quantitative (or quantitative + qualitative) (93%) vs. qualitative only, and observational (57%) vs. experimental.

**Table 3:** Study Design and Sample Information by Number and Percent of Articles

		Total Articles* n*=150		
		*n*		%
*STUDY LOCATION				
Canada		4	2.67	
China		12	8.00	
France		6	4.00	
Germany		24	16.00	
Iran		5	3.33	
Ireland		3	2.00	
Japan		5	3.33	
Korea		5	3.33	
Netherlands		13	8.67	
Norway		7	4.67	
Singapore		3	2.00	
Spain		8	5.33	
Sweden		4	2.67	
United Kingdom		3	2.00	
**United States**		**28**	**18.67**	
Other (<3 articles)		21	14.00	
STUDY DESIGN				
	Cross-Sectional	50	33.33	
	**Longitudinal**	**100**	**66.67**	
	Qualitative	11	7.33	
	**Quantitative**	**134**	**89.33**	
	Qualitative + Quantitative	5	3.33	
	Experimental	64	42.67	
	**Observational**	**86**	**57.33**	
SAMPLE SIZE				
	< 100	63	42.00	
	**≥ 100**	**85**	**56.67**	
	Reporting Discrepancy	2	1.33	
AGE (Range *or* Mean ± SD in Years)				
	**Reported**	**103**	**68.67**	
	Not Reported	47	31.33	
RACE				
	Reported	27	18.00	
	**Not Reported**	**123**	**82.00**	
*CANCER DIAGNOSIS				
Blood Dyscrasias		35	23.33	
Bone Cancer		8	5.33	
**Breast Cancer**		**119**	**79.33**	
CNS Malignancies		8	5.33	
GI Malignancies		53	35.33	
GU Malignancies		49	32.67	
Head & Neck Cancer		15	10.00	
Lung Cancer		26	17.33	
Neuroendocrine Tumors		1	0.67	
Skin Cancer		7	4.67	
Smooth Muscle Tumors		1	0.67	
Unspecified		27	18.00	
*CANCER TREATMENT (Obs. Studies *n*=86)				
	**Chemotherapy**	**69**	**80.23**	
	Hormonal Therapy	33	38.37	
	Immunotherapy	8	9.30	
	PARP Inhibitors	1	1.16	
	Radiotherapy	51	59.30	
	Surgery	47	54.65	
	Other	13	15.12	
*CANCER-RELATED FATIGUE INTERVENTION (Exp. Studies *n*=64)				
	**Integrative Medicine**	**29**	**45.31**	
	Physical Exercise	26	40.63	
	Psychosocial Intervention	12	18.75	

ALL CAPS: the study variables

**Bolded** font: the most common subcategory for each variable

Abbreviations: CNS=Central Nervous System, GI=Gastrointestinal, GU=Genitourinary,

Obs.=Observational, Exp.=Experimental

Variables marked with an asterisk (*) indicate that some articles contained multiple subcategories, so the sum of the subcategory numbers and percentages exceed 100% for these variables. In addition, the “Other” subcategory under Study Location indicates countries with fewer than 3 articles, which included: Brazil *n*=2, Cyprus *n*=2, Jordan *n*=2, Turkey *n*=2, Belgium* n*=1, Bulgaria *n*=1, Denmark *n*=1, India *n*=1, Indonesia *n*=1, Israel *n*=1, Italy *n*=1, Malaysia *n*=1, New Zealand *n*=1, Switzerland *n*=1, Taiwan *n*=1, Thailand *n*=1, Unknown *n*=1

## Discussion

CRF is one of the most common, distressing conditions experienced by people with cancer. It has detrimental impacts on daily functioning and overall quality of life [19, 20]. The absence of an accepted definition and standardized approach for assessing CRF has contributed to the heterogeneity of methods and results across published studies. Synthesizing the current literature in adults with cancer that includes CRF as a primary outcome is essential to identify gaps in the literature and provide recommendations for future research to improve health outcomes in adults with cancer. This scoping review identified, collected, and summarized information to: 1) describe how CRF has been defined, 2) determine how CRF has been assessed, and 3) characterize the articles and samples reviewed. Out of 150 included articles, CRF definitions and methodological procedures (e.g., study designs, clinical measures and dimensions) varied widely, confirming the need for a single agreed-upon CRF definition and assessment battery.

### How CRF was defined

Consensus has not been reached regarding how to define CRF, as reflected in this review in which only two-thirds of the articles defined/described CRF. Less than half of the articles that defined/described CRF used the NCCN’s definition, and the articles with non-NCCN definitions/descriptions were highly heterogeneous, providing further evidence that the field is lacking consensus on how to define or conceptualize CRF. As the debate continues whether CRF is a definable condition, the need to use a consistent definition is critical to translate research findings to the clinical setting and accurately describe, diagnose, and manage CRF.

Of the articles that contained definitions/descriptions, the majority included the word ‘multidimensional’ and/or listed more than one dimension. Some authors may have avoided using the term multidimensional to describe CRF because it is still debated whether CRF should be considered a multidimensional construct (in which all dimensions share the same etiology) or a unidimensional construct (in which each dimension has a stand-alone pathogenesis) [21, 22]. In addition, all dimensions that end up being included in the agreed-upon definition should also be operationalized to solidify a collective understanding of the construct of CRF.

A recent review of CRF among *childhood* cancer survivors formulated an explicit definition based on the included reports, defining it as “a subjective, persistent, and multidimensional experience that differs from normal fatigue in the physical, emotional and/or cognitive spheres [23].” Conducting a similar thematic review in adults with CRF may be helpful to establish the groundwork for an explicit definition. In addition, creating research-based case definitions of CRF and delineating specific clinical subtypes may assist with understanding CRF’s etiology and formulating optimal management strategies; this was a recommendation made during the National Cancer Institute Clinical Trials Planning Meeting in 2013 [24] that is still unmet.

### How CRF was assessed

In this review, we found that nearly 30 measures have been used to assess CRF, with each measure differing widely in scope, including varying numbers of items, scale formats (e.g., visual analogue scales, Likert ratings), scoring interpretations, and reference periods for which CRF was assessed (e.g., present day, past week). The MFI-20 was the CRF measure used most often; the same questionnaire was also the most commonly used in our previous review of fatigue in non-oncologic conditions [25], perhaps because it broadly measures general fatigue as well as specific fatigue dimensions like physical, motivational, cognitive, and mental fatigue. In the current review, only about half of the articles included validity descriptions with their measures; it’s important for this information to be reported in future publications so that readers know whether the tool is validated in the specific cancer population being examined, and they can use that information when interpreting the results. Across all CRF measures, 30 unique dimensions were found, with some dimensions used more heavily (“Physical”, “Mental”, “Cognitive”) than others, perhaps based on the most common symptoms reported by a specific cancer type (i.e., breast cancer).

The current number of available tools and their heterogeneity pose a challenge for comparing and interpreting CRF findings across articles. An additional challenge is that each tool assesses unique dimensions of CRF, and these dimensions are not operationalized. Therefore, it is imperative for the field to either identify which existing CRF measure(s) sufficiently encompass the breadth of CRF as a behavioral construct or create a new CRF measure (or battery) to move toward a standardized way of assessing CRF. In doing so, the number of dimensions used to measure CRF will need to be reduced, and each dimension will need to be operationalized.

Many articles included in this review failed to provide accurate and/or complete information regarding measures used to assess CRF, including: (1) the correct name and/or citation of the assessment(s), (2) the version of the tool (e.g., short form vs. long form, English vs. French), and (3) interpretation of scores in relation to CRF. These reporting inconsistencies/omissions led us to collapse all versions of a measure into the same group with the original measure and describe the psychometric properties of only the original versions. In the future, authors must report CRF measure information in full (i.e., proper name, correct citation, version, scoring interpretation) so that consumers can confidently determine whether measures used across articles are identical and interpret findings accordingly.

### Characteristics of included studies

CRF was primarily examined in high-income countries, in that half of the studies were conducted in either the United States, Germany, Netherlands, or China**.** A significant portion (43%) of articles contained small sample sizes (fewer than 100 people). Sample demographics were grossly underreported, with missing data commonly occurring for race, age, and gender; based on what *was* reported, samples primarily consisted of White middle-aged females. These small homogenous samples limit the ability to translate findings to the broader population, impeding the development of effective therapies. In future studies, demographic information is crucial to report because these characteristics can impact cancer-related symptoms. For example, those who are female [26] or of older age [27, 28] have been found to experience higher levels of CRF. Demographic factors that are considered social determinants of health are particularly important to include in publications because certain groups of people experience disproportionately poorer access or outcomes related to cancer care due to structural disparities and inequities [29].

Cancer type was unspecified in one-sixth of the articles. Breast cancer was the most common cancer type in which CRF was evaluated, which is not surprising since breast cancer was the number one cancer diagnosis world-wide as of 2020 [30]. The most common cancer treatment (observational articles only) was chemotherapy, likely because it is a common primary treatment for invasive breast cancer and its behavioral toxicities are well-documented [31, 32]. It was encouraging to find that longitudinal designs were more common than cross-sectional, suggesting that the long-term implications of CRF have been considered and evaluated throughout the course of disease; similarly, quantitative designs were more common than qualitative, suggesting that authors have stived to obtain objective, reliable, and generalizable results. However, observational designs were more common than experimental, likely due to the need to observe the natural trajectory of CRF as a late side effect of most cancer treatments. Although observational study designs may be the only way researchers can explore certain questions, they only allow us to determine *associations* with CRF (not causality) and typically contain more uncontrolled confounds, limiting the interpretations we can yield from the findings.

### Limitations of this review

Although an extensive review of the literature was conducted, it is possible this review may have missed relevant articles due to the selected search terms or applied filters. Regarding search terms, CRF was challenging to operationalize due to the number of words that are synonymous with fatigue, so we opted to be as specific as possible with the words used to describe CRF (see Online Resource 2) and left out more general fatigue-related terms (e.g., lethargy, weakness). As for applied filters, we only included articles written in or translated to English, so the findings here may be more applicable to English-speaking countries and not truly representative of CRF studies worldwide. Lastly, despite our best intentions to do so, our review did not include the dimensions included in CRF definitions; this is because many of the articles provided general descriptions rather than true definitions, and it was unclear whether these descriptions were being used to operationalize the term for the study or merely re-count how it has been described in previous literature.

## Conclusion

Evidence from this scoping review highlights the heterogeneity of methods used across articles in adults with cancer-related fatigue that have resulted from the *absence* of a single agreed-upon definition for CRF and consensus on which assessments most accurately measure CRF and its dimensions. The methodological variability across CRF studies limits our potential to help alleviate or prevent CRF symptoms, so it is essential for the field to agree on how to define CRF, identify the clinical assessment(s) that should be used to measure CRF, and operationalize the dimensions used in the definition and assessment of CRF.

### Supplementary Information

Below is the link to the electronic supplementary material.

Supplementary file1 (DOCX 38 KB)

Supplementary file2 (DOCX 42 KB)

Supplementary file3 (DOCX 48 KB)

Supplementary file4 (XLSX 48 KB)

Supplementary file5 (XLSX 95 KB)

## Data Availability

All data supporting the findings of this study are available within the paper and its Supplementary Information.
