# Supplementary material for: The definitions, assessment, and dimensions of cancer-related fatigue: A scoping review
Source: Support Care Cancer. 2024 Jun 25;32(7):457. doi: 10.1007/s00520-024-08615-y (PMC11199267; doi:10.1007/s00520-024-08615-y)
Supplement: Supplementary file 1 — Supplementary file1 (DOCX 38 KB) [file 520_2024_8615_MOESM1_ESM.docx]

|  | **INCLUSION CRITERIA**  (ALL criteria must be true to pass) | **EXCLUSION CRITERIA**  (at least ONE criterion must be true to exclude) |
| --- | --- | --- |
| TITLE & ABSTRACT SCREENING | |  |
| *Article passes criteria if:* | | *Article fails criteria if:* |
|  | 1. Record contains an abstract | 1. Record does not contain an abstract |
|  | 2. Publication type is full-length original research  *Note: pass article to full-text stage if this information cannot be discerned but article meets all other inclusion criteria* | 2. Publication type is a thesis or dissertation **OR**  is not full-length original research, such as:   - Brief report / case report / case study / concise report / short communication / short report - Clinical education - Comment / correspondence / letter to editor - Commentary / editorial / opinion / perspective /   special report   - Conference abstract / proceeding / series - Consensus statement - Data drawn from secondary sources - Drug trial not studying cancer-related fatigue treatment - Event description - Initial assessment - Literature search / meta-analysis / review - Modeling study - Personal reflection / story - Pilot / preliminary study - Position statement - Study protocol |
|  | 3. Publication year is between 2010 and 2020 | 3. Publication year is before 2010 or after 2020 |
|  | 4. Publication language is English | 4. Publication language is not English |
|  | 5. Participant sample:   - Includes humans in-vivo **AND** - Has a cancer diagnosis, as defined by <https://www.cancer.gov/types> **AND** - Includes adults only (18+ years)   *Note: pass article to full-text stage if this information cannot be discerned but article meets all other inclusion criteria* | 5. Participant sample:   - Does not include humans in-vivo **OR** - Does not include a group with cancer **OR** - Includes children **OR** - Is a mixed sample (not only patients with cancer) **OR** - Includes dyads & triads (e.g., patients, caregivers) **OR** - Includes animals **OR** - Includes in-vitro |
|  | 6. Cancer-related fatigue:   - Is the primary outcome (explicitly mentioned in   methods/results) **AND**   - Is described in terms of how it was assessed **AND** - Is defined/described/assessed as a dimensional construct   *Note: pass article to full-text stage if this information cannot be discerned but article meets all other inclusion criteria* | 6. Cancer-related fatigue:   - Is not the primary outcome **OR** - Is not described in terms of how it was assessed **OR** - Is not defined/described/assessed as a dimensional construct **OR** - Is not in the correct context (e.g., compassion fatigue, general fatigue, occurs as an adverse event, results from toxicity) |
| FULL-TEXT SCREENING | |  |
| *Article passes criteria if:* | | *Article fails criteria if:* |
|  | 1. Able to access the full-text article | 1. Not able to access the full-text article |
|  | 2. Publication language is English | 2. Publication language is not English |
|  | 3. Cancer-related fatigue is the primary outcome  (explicitly mentioned in methods/results) | 3. Cancer-related fatigue is not the primary  outcome |
|  | 4. Publication type is full-length original research | 4. Publication type is a thesis or dissertation **OR**  is not full-length original research, such as:   - Brief report / case report / case study / concise report / short communication / short report - Clinical education - Comment / correspondence / letter to editor - Commentary / editorial / opinion / perspective /   special report   - Conference abstract / proceeding / series - Consensus statement - Data drawn from secondary sources - Drug trial not studying cancer-related fatigue treatment - Event description - Initial assessment - Literature search / meta-analysis / review - Modeling study - Personal reflection / story - Pilot / preliminary study - Position statement - Study protocol |
|  | 5. Participant sample consists of *only* patients with cancer | 5. Participant sample includes mixed groups (participants who did not have cancer) |
|  | 6. Cancer-related fatigue:   - Is described in terms of how it was assessed **AND** - Is defined/described/assessed as a dimensional construct | 6. Cancer-related fatigue:   - Is not described in terms of how it was assessed **OR** - Is not defined/described/assessed as a dimensional construct **OR** - Is not in the correct context (e.g., compassion fatigue, general fatigue, occurs as an adverse event, results from toxicity) |
|  | 7. Participant sample:   - Humans in-vivo **AND** - Has a cancer diagnosis, as defined by <https://www.cancer.gov/types> **AND** - Adults (18+ years) | 7. Participant sample:   - Does not include humans in-vivo **OR** - Does not include a group with cancer **OR** - Includes children **OR** - Includes animals **OR** - Includes in-vitro |
|  |  |  |

Title: The definitions, assessment, and dimensions of cancer-related fatigue: A scoping review

Journal: *Supportive Care in Cancer*

Authors: Kayla F. Keane, Jordan Wickstrom, Alicia A. Livinski, Catherine Blumhorst, Tzu-fang Wang, Leorey N. Saligan

Corresponding Author Name, Affiliation, and Email: Leorey N. Saligan; National Institute of Nursing Research, National Institutes of

Health, Bethesda, MD, USA; [Leorey.Saligan@nih.gov](mailto:Leorey.Saligan@nih.gov)
