# Supplementary material for: The definitions, assessment, and dimensions of cancer-related fatigue: A scoping review
Source: Support Care Cancer. 2024 Jun 25;32(7):457. doi: 10.1007/s00520-024-08615-y (PMC11199267; doi:10.1007/s00520-024-08615-y)
Supplement: Supplementary file 2 — Supplementary file2 (DOCX 42 KB) [file 520_2024_8615_MOESM2_ESM.docx]

**Database:** PubMed
**Platform:** US National Library of Medicine
**Date Searched:** January 25, 2021
**Date Limits:** January 1, 2010–December 31, 2020
**Other Limits / Filters:** English; Human; Excludes: case report, editorial, commentary, conference abstracts, letter, retraction, reviews

| **Set** | **Concept** | **Search Strategy** |
| --- | --- | --- |
| #1 | Cancer Related | (“cancer survivor*”[tiab] OR “cancer patient*”[tiab] OR “oncology patient*”[tiab] OR “oncology survivor*”[tiab] OR “cancer survivorship”[tiab] OR “oncology survivorship”[tiab] OR "Cancer Survivors"[Majr]) |
| #2 | Fatigue | (fatigue[tiab] OR fatigues[tiab] OR fatigued[tiab] OR fatiguing[tiab] OR tiring[tiab] OR tired[tiab] OR tiredness[tiab] OR exhaustion[tiab] OR exhausted[tiab] OR weariness[tiab] OR Fatigue[Majr]) |
| #3 |  | #1 AND #2 |
| #4 | Limits & Filters | #3 AND (("2010/01/01"[Date - Publication] : "2020/12/31"[Date - Publication])) AND (english[lang]) |
| #5 |  | #4 NOT (("Animals"[Mesh] NOT ("Animals"[Mesh] AND "Humans"[Mesh])) NOT (mice[tiab] OR mouse[tiab] OR rat[tiab] OR rats[tiab] OR rodent*[tiab] OR animal*[tiab]) |
| #6 |  | #5 NOT (letter[ptyp] OR editorial[ptyp] OR comment[ptyp] OR news[ptyp] OR "Congress"[Publication Type] OR "Consensus Development Conference"[Publication Type] OR "Review"[Publication Type] OR "Systematic Review"[Publication Type] OR “meta-analysis”[ptyp] OR “retracted publication”[ptyp] OR “retraction of publication”[ptyp] OR "Published Erratum"[Publication Type] OR "Case Reports" [Publication Type] OR “systematic review*”[ti] OR “meta-analysis”[ti] OR “meta-analyses”[ti] OR “retraction notice”[ti] OR protocol[ti] OR protocols[ti] OR “case report”[ti] OR “case series”[ti] OR letter[ti] OR editorial*[tiab] OR commentary[tiab] OR “conference abstract*”[tiab] OR “conference proceeding*”[tiab] OR “conference paper*”[tiab] OR “retraction of publication”[tiab] OR “retracted publication”[tiab] OR corrigenda[tiab] OR corrigendum[tiab] OR errata[tiab] OR erratum[tiab]) |

**Database:** Embase
**Platform:** Elsevier
**Date Searched:** January 25, 2021
**Date Limits:** 2010–2020
**Other Limits / Filters:** English; Source: Embase; Human; Excludes: case report, editorial, commentary, conference abstracts, letter, retraction, reviews

| **Set** | **Concept** | **Search Strategy** |
| --- | --- | --- |
| #1 | Cancer Related | ('cancer survivor*':ti,ab OR 'cancer patient*':ti,ab OR 'oncology patient*':ti,ab OR 'oncology survivor*':ti,ab OR 'cancer survivorship':ti,ab OR 'oncology survivorship':ti,ab OR 'cancer survivor'/mj OR 'cancer patient'/mj) |
| #2 | Fatigue | (fatigue:ti,ab OR fatigues:ti,ab OR fatigued:ti,ab OR fatiguing:ti,ab OR tiring:ti,ab OR tired:ti,ab OR tiredness:ti,ab OR exhaustion:ti,ab OR exhausted:ti,ab OR weariness:ti,ab OR 'fatigue'/mj OR 'cancer fatigue'/mj) |
| #3 |  | #1 AND #2 |
| #4 | Limits & Filters | #3 AND ([embase]/lim OR [embase classic]/lim) AND [english]/lim AND [2010-2020]/py |
| #5 |  | #4 NOT ([animals]/lim NOT ([animals]/lim AND [humans]/lim))  NOT (mice:ti,ab OR mouse:ti,ab OR rat:ti,ab OR rats:ti,ab OR rodent*:ti,ab OR animal*:ti,ab) |
| #6 |  | #5 NOT ([conference abstract]/lim OR [conference paper]/lim OR [conference review]/lim OR [data papers]/lim OR [editorial]/lim OR [erratum]/lim OR [letter]/lim OR [note]/lim OR [review]/lim OR [short survey]/lim OR [systematic review]/lim OR [meta analysis]/lim OR 'conference abstract'/exp OR 'conference paper'/exp OR 'data paper'/exp OR 'editorial'/exp OR 'letter'/exp OR 'erratum'/exp OR 'retraction notice'/exp OR 'note'/exp OR 'short survey'/exp OR 'review'/exp OR 'systematic review'/exp OR 'case report'/exp OR 'case study'/exp OR “case report*”:ti OR “case series”:ti OR “systematic review*”:ti OR “meta analysis”:ti OR “meta analyses”:ti OR “retraction notice”:ti OR protocol:ti OR protocols:ti OR letter:ti OR corrigenda:ti,ab OR corrigendum:ti,ab OR errata:ti,ab OR erratum:ti,ab OR editorial*:ti,ab OR commentary:ti,ab OR “conference abstract*”:ti,ab OR “conference proceeding*”:ti,ab OR “conference paper*”:ti,ab OR “retraction of publication”:ti,ab OR “retracted publication”:ti,ab) |

**Database:** CINAHL Plus
**Platform:** EBSCOhost
**Date Searched:** January 25, 2021
**Date Limits:** 2010–2020
**Other Limits / Filters:** English; Source: Embase; Human; Excludes: case report, editorial, commentary, conference abstracts, letter, retraction, reviews

| **Set** | **Concept** | **Search Strategy** |
| --- | --- | --- |
| #1 | Cancer Related | Title: (“cancer survivor” OR “cancer survivors” OR “cancer patient” OR “cancer patients” OR “oncology patient” OR “oncology patients” OR “oncology survivor” OR “oncology survivors” OR “cancer survivorship” OR “oncology survivorship”) |
| #2 | Cancer Related | Abstract: (“cancer survivor” OR “cancer survivors” OR “cancer patient” OR “cancer patients” OR “oncology patient” OR “oncology patients” OR “oncology survivor” OR “oncology survivors” OR “cancer survivorship” OR “oncology survivorship”) |
| #3 | Cancer Related | Exact Major Subject Heading: (MM "Cancer Patients") OR (MM "Cancer Survivors") |
| #4 |  | #1 OR #2 OR #3 |
| #5 | Fatigue | Title: (fatigue OR fatigues OR fatigued OR fatiguing OR tiring OR tired OR tiredness OR exhaustion OR exhausted OR weariness) |
| #6 | Fatigue | Abstract: (fatigue OR fatigues OR fatigued OR fatiguing OR tiring OR tired OR tiredness OR exhaustion OR exhausted OR weariness) |
| #7 | Fatigue | Exact Major Subject Heading: (MM "Cancer Fatigue") OR (MM "Fatigue") |
| #8 |  | #5 OR #6 OR #7 |
| #9 |  | #4 AND #8 |
| #10 | Limits & Filters | Title: (protocol OR protocols OR "case report*" OR “case series” OR "retraction notice" OR "systematic review*" OR "meta analysis" OR “meta analyses” OR letter OR editorial* OR "conference abstract*" OR "conference paper*" OR “conference proceeding*” OR commentary OR corrigenda OR corrigendum OR errata OR erratum OR “retraction of publication” OR “retracted publication”) |
| #11 | Limits & Filters | Abstract: (protocol OR protocols OR "case report*" OR “case series” OR "retraction notice" OR "systematic review*" OR "meta analysis" OR “meta analyses” OR letter OR editorial* OR "conference abstract*" OR "conference paper*" OR “conference proceeding*” OR commentary OR corrigenda OR corrigendum OR errata OR erratum OR “retraction of publication” OR “retracted publication”) |
| #12 | Limits & Filters | Exact Subject Heading: (MH "Case Studies") OR (MH "Systematic Review") OR (MH "Scoping Review") OR (MH "Literature Review") OR (MH "Meta Analysis") OR (MH "News") OR (MH "Retraction of Publication") OR (MH "Retracted Publication") OR (MH "Theses and Dissertations") |
| #13 |  | #10 OR #11 OR #12 |
| #14 | Limits & Filters | Exact Subject Heading: ((MH "Animals, Laboratory") OR (MH "Rodents+") OR (MH "Mice") OR (MH "Rats")) |
| #15 | Limits & Filters | Exact Subject Heading: ((MH "Animals+") NOT ((MH "Human") AND (MH "Animals+"))) |
| #16 | Limits & Filters | Title: (mice OR mouse OR rat OR rats OR rodent* OR animal*) |
| #17 | Limits & Filters | Abstract: NOT (mice OR mouse OR rat OR rats OR rodent* OR animal*) |
| #18 | Limits & Filters | #14 OR #15 OR #16 OR #17 |
| #19 |  | #13 NOT #18 |
| #20 |  | #19 AND Language: English AND Publication Year: 2010–2020 |

**Database:** PsycNet: PsycINFO & PsycARTICLES
**Platform:** American Psychological Association
**Date Searched:** January 25, 2021
**Date Limits:** 2010–2020
**Other Limits / Filters:** English; Human; Excludes: case report, editorial, commentary, conference abstracts, letter, retraction, reviews

| **Set** | **Concept** | **Search Strategy** |
| --- | --- | --- |
| #1 | Cancer Related | MeSH: {Cancer Survivors} |
| #2 | Cancer Related | Title: (“cancer survivor” OR “cancer survivors” OR “cancer patient” OR “cancer patients” OR “oncology patient” OR “oncology patients” OR “oncology survivor” OR “oncology survivors” OR “cancer survivorship” OR “oncology survivorship”) |
| #3 | Cancer Related | Abstract: (“cancer survivor” OR “cancer survivors” OR “cancer patient” OR “cancer patients” OR “oncology patient” OR “oncology patients” OR “oncology survivor” OR “oncology survivors” OR “cancer survivorship” OR “oncology survivorship”) |
| #4 |  | #1 OR #2 OR #3 |
| #5 | Fatigue | Index Term: {Fatigue} OR MesH: {Fatigue} |
| #6 | Fatigue | Title: (fatigue OR fatigues OR fatigued OR fatiguing OR tiring OR tired OR tiredness OR exhaustion OR exhausted OR weariness) |
| #7 | Fatigue | Abstract: (fatigue OR fatigues OR fatigued OR fatiguing OR tiring OR tired OR tiredness OR exhaustion OR exhausted OR weariness) |
| #8 |  | #5 OR #6 OR #7 |
| #9 |  | #4 AND #8 |
| #10 | Limits & Filters | #9 AND Language: English AND Publication Year: 2010–2020 |
| #11 | Limits & Filters | Index Terms: {Systematic Review} OR {Meta Analysis} OR {Literature Review} OR {Case Report} |
| #12 | Limits & Filters | Abstract: (protocol OR protocols OR "case report*" OR “case series” OR "retraction notice" OR "systematic review*" OR "meta analysis" OR “meta analyses” OR letter OR editorial* OR "conference abstract*" OR "conference paper*" OR “conference proceeding*” OR commentary OR corrigenda OR corrigendum OR errata OR erratum OR “retraction of publication” OR “retracted publication”) |
| #13 | Limits & Filters | Title: (protocol OR protocols OR "case report*" OR “case series” OR "retraction notice" OR "systematic review*" OR "meta analysis" OR “meta analyses” OR letter OR editorial* OR "conference abstract*" OR "conference paper*" OR “conference proceeding*” OR commentary OR corrigenda OR corrigendum OR errata OR erratum OR “retraction of publication” OR “retracted publication”) |
| #14 |  | #11 OR #12 OR #13 |
| #15 |  | #10 NOT #14 |
| #16 | Limits & Filters | Index Terms: NOT {Mice} OR {Rodents} OR {Mice} OR {Rats} |
| #17 | Limits & Filters | Title: (mice OR mouse OR rat OR rats OR rodent* OR animal*) |
| #18 | Limits & Filters | Abstract: (mice OR mouse OR rat OR rats OR rodent* OR animal*) |
| #19 |  | #16 OR #17 OR #18 |
| #20 |  | #15 NOT #19 |
| #21 |  | #20 AND Publication Type: “Peer Reviewed Journal” |

Title: The definitions, assessment, and dimensions of cancer-related fatigue: A scoping review

Journal: *Supportive Care in Cancer*

Authors: Kayla F. Keane, Jordan Wickstrom, Alicia A. Livinski, Catherine Blumhorst, Tzu-fang Wang, Leorey N. Saligan

Corresponding Author Name, Affiliation, and Email: Leorey N. Saligan; National Institute of Nursing Research, National Institutes of

Health, Bethesda, MD, USA; [Leorey.Saligan@nih.gov](mailto:Leorey.Saligan@nih.gov)
