# Supplementary material for: The definitions, assessment, and dimensions of cancer-related fatigue: A scoping review
Source: Support Care Cancer. 2024 Jun 25;32(7):457. doi: 10.1007/s00520-024-08615-y (PMC11199267; doi:10.1007/s00520-024-08615-y)
Supplement: Supplementary file 3 — Supplementary file3 (DOCX 48 KB) [file 520_2024_8615_MOESM3_ESM.docx]

**General Information**

**Journal**

**Title of Article**

Copy/paste verbatim from article

**First Author**

Copy/paste verbatim from article

**Year of Official Publication (not e-publication)**

**Country Study was Conducted (leave blank if not reported)**

Select all countries in which the study was conducted (likely found in Methods/Introduction section); this is NOT the same as author affiliation

1. United States
2. France
3. Canada
4. China
5. Germany
6. Jordan
7. Korea
8. Netherlands
9. Sweden
10. Other

**Methodology**

**Study Design Pt. 1**

Select “cross sectional” OR “longitudinal”

Select “qualitative” AND/OR “quantitative” (select both qual/quant if mixed-methods study)

1. Cross sectional

1. Longitudinal

1. Qualitative

1. Quantitative

**Study Design Pt. 2**

Select “observational” if no intervention beyond regular care; select “experimental” if fatigue-specific intervention

(e.g., impact of cognitive behavioral therapy on cancer symptoms)

1. Observational

1. Experimental

**Cancer Treatment (observational studies only)**

List name of cancer treatment(s) verbatim (e.g., chemotherapy, surgery, radiation, normative palliative care, immunotherapy, hormone therapy)

**Cancer Intervention (experimental studies only)**

List name of intervention(s) verbatim (e.g., yoga, cognitive behavioral therapy, high- and low-intensity exercise). Do not include details about dosage, duration, or frequency

**Participants - Extract from Sociodemographic Table (if possible)**

**Number of Participants Overall**

Enter sample size used for data analysis (final N after dropout)

**Number of Participants by Gender**

Enter sample size used for each gender category: e.g., male (10), female (10)

**Sample Age (Range OR Mean Age)**

Enter age range using this format: 18-55 (priority is age range). Only extract mean age if range not reported

**Sample Race Categories**

Copy/paste verbatim from article

**Cancer Diagnosis**

List each cancer diagnosis/type included in the sample

**CRF Characteristics**

**Definition of CRF**

Indicate whether CRF is described as multidimensional in the paper (and/or multiple dimensions of CRF are listed)

1. Yes
2. No
3. Definition not provided (not reported)

Indicate whether the article contains the NCCN definition of CRF, “Cancer-related fatigue is a distressing, persistent, subjective sense of physical, emotional, and/or cognitive tiredness or exhaustion related to cancer or cancer treatment that is not proportional to recent activity and interferes with usual functioning”

1. Yes
2. No
3. Definition not provided (not reported)

**CRF Dimensions Assessed in Study (Quantitative Studies Only)**

Indicate whether CRF is assessed using a multidimensional measure

1. Yes
2. No

Select all dimensions provided in the Results section. If exact word not on list, please select "other" and fill in verbatim. Careful: if tool assesses multiple dimensions of fatigue but authors only assess one dimension, ONLY select that one

1. Physical

1. Mental

1. Motivational

1. Affective

1. Emotional

1. Cognitive

1. Behavioral

1. Sensory

1. Other

**CRF Measures**

MEASURE: Use format: name of tool (acronym) (e.g. "Attentional Fatigue Index (AFI)"; "focus group interview")

VALIDATED: Use format: "mentioned" OR "not mentioned"

VALIDATION DESCRIPTION: copy verbatim if mentioned whether measure is validated

TIMEPOINT: For cross-sectional studies, indicate whether CRF was measured before, during, or after primary treatment, or if this information was not reported. Use format: "Before", "During", "After", "Mixed", “Not Reported”

| **Measure**  **Name** | **Validated**  ***(mentioned or not mentioned)*** | **Validation description**  ***(if applicable)*** | **Timepoint**  ***(only if cross-sectional)*** |
| --- | --- | --- | --- |
|  |  |  |  |

**Outcomes Related to CRF Dimensions**

Include 1-2 sentences for each dimension verbatim from article

Quantitative: For each dimension, extract whether CRF changed significantly (+ p-value) and whether dimensions were linked to sociodemographic/biological characteristic (i.e.: hemoglobin, sex, race, etc.). Use parentheses to note the table/figure results can be found

Qualitative: extract themes related to CRF dimensions – include quotes. Use parentheses to denote your interpretations

Title: The definitions, assessment, and dimensions of cancer-related fatigue: A scoping review

Journal: *Supportive Care in Cancer*

Authors: Kayla F. Keane, Jordan Wickstrom, Alicia A. Livinski, Catherine Blumhorst, Tzu-fang Wang, Leorey N. Saligan

Corresponding Author Name, Affiliation, and Email: Leorey N. Saligan; National Institute of Nursing Research, National Institutes of

Health, Bethesda, MD, USA; [Leorey.Saligan@nih.gov](mailto:Leorey.Saligan@nih.gov)
